# Within-day dynamics of plant–pollinator networks are dominated by early flower closure: an experimental test of network plasticity

**DOI:** 10.1007/s00442-021-04952-5

**Published:** 2021-06-03

**Authors:** Benjamin Schwarz, Carsten F. Dormann, Diego P. Vázquez, Jochen Fründ

**Affiliations:** 1 Biometry and Environmental System Analysis, University of Freiburg, Tennenbacher Str. 4, 79106 Freiburg, Germany; 2 Argentine Institute for Dryland Research, CONICET, Av. Ruiz Leal s/n, 5500 Mendoza, Argentina; 3 Faculty of Exact and Natural Sciences, National University of Cuyo, Padre Jorge Contreras 1300, M5502JMA Mendoza, Argentina

**Keywords:** Cichorieae, Temporal turnover, Diel dynamics, Circadian rhythms, Flower visitation

## Abstract

Temporal variability of plant–pollinator interactions is important for fully understanding the structure, function, and stability of plant–pollinator networks, but most network studies so far have ignored within-day dynamics. Strong diel dynamics (e.g., a regular daily cycle) were found for networks with Cichorieae, which typically close their flowers around noon. Here, we experimentally prevented early flower closure to test whether these dynamics are driven by the temporally limited availability of Cichorieae, or by timing of pollinator activity. We further tested if the dynamics involving Cichorieae and their pollinators also affect the dynamics on other plants in the network. Finally, we explored the structure of such manipulated networks (with Cichorieae available in the morning and afternoon) compared to unmanipulated controls (Cichorieae available only in the morning). We found that flower closure of Cichorieae is indeed an important driver of diel network dynamics, while other drivers of pollinator timing appeared less important. If Cichorieae flowers were available in the afternoon, they were visited by generalist and specialist pollinators, which overall decreased link turnover between morning and afternoon. Effects of afternoon availability of Cichorieae on other plants in the network were inconclusive: pollinator switching to and from Cichorieae tended to increase. On the level of the aggregated (full-day) network, the treatment resulted in increased dominance of Cichorieae, reducing modularity and increasing plant generality. These results highlight that network dynamics can be predicted by knowledge of diel or seasonal phenology, and that fixed species timing assumptions will misrepresent the expected dynamics.

**Supplementary Information:**

The online version contains supplementary material available at 10.1007/s00442-021-04952-5.

## Introduction

Temporal dynamics of plant–pollinator networks arise due to both phenological turnover of species and switching of interaction partners over time. In recent years, an increasing number of studies have emphasized that considering such temporal dynamics appears to be pivotal for our understanding of the structure, function, and stability of plant–pollinator networks (Burkle and Alarcón 2011; Trøjelsgaard and Olesen 2016; Schwarz et al. 2020; CaraDonna et al. 2021). For example, the phenological turnover of species introduces temporally forbidden links into networks and thereby constrains network structure (Vázquez et al. 2009; Olesen et al. 2011), while switching of interaction partners over time (i.e., link rewiring) may indicate that networks are inherently resilient to changes in species composition (CaraDonna et al. 2017). In addition, a temporally explicit perspective can increase our understanding of indirect interactions within plant–pollinator networks (Baldock et al. 2011; Rasmussen et al. 2013). However, our knowledge of the temporal dynamics of plant–pollinator networks is primarily based on seasonal dynamics of plant–pollinator interactions (CaraDonna et al. 2021). Network dynamics on daily time scales have rarely been considered (but see: Baldock et al. 2011; Fründ et al. 2011; Bloch et al. 2017; Kronfeld-Schor et al. 2017) despite awareness of the importance of diel changes for plant–pollinator interactions (Willmer and Corbet 1981; Herrera 1990; Willmer and Stone 2004).

Several drivers of diel temporal dynamics of plant–pollinator interactions have been identified, including internal rhythms, environmental conditions and availability of interaction partners. Plants and pollinators exhibit circadian rhythms controlled by internal clocks, which may be entrained by light and temperature cues (Bloch et al. 2017; Fenske et al. 2018). In addition, environmental factors may further constrain the timing of plants and pollinators. For example, temperature is one of the main drivers of abundance and species composition of flower visitors within a day (McCall and Primack 1992; Totland 1994; Rader et al. 2013; Knop et al. 2018). Herrera (1990) suggested that diel dynamics of plant–pollinator interactions are driven by independent daily cycles of plants and pollinators, with pollinators responding to environmental factors rather than to the quantity and quality of floral resources. Supporting this view, some pollinators were unable to immediately respond to subtle changes in nectar production (Fowler et al. 2016), and even to experimentally prolonged nectar availability (Gottlieb et al. 2005). However, in other cases plants and pollinators appear to flexibly respond to changes in the other trophic level (Spiesman and Gratton 2016), suggesting that the temporal activity of pollinators may indeed respond to diel variation in floral resources (Stone et al. 1999). Timing of flowers can also change in response to pollinators: in some plant species the timing of flower closure within a day can be adjusted depending on pollen deposition (Fründ et al. 2011), while nectar replenishment may be triggered by nectar consumption (Castellanos et al. 2002).

Such plasticity of plant–pollinator interactions may be critical for the functioning of networks at daily time scales. Flexibility in timing and interaction partners may guarantee that plants receive sufficient pollination services and that pollinators meet their energy requirements throughout the day. In addition, timing and flexibility of interactions are relevant for indirect effects between species of the same trophic level that are coupled by common interaction partners (apparent competition and facilitation). For example, the presence of plant species attractive to pollinators was found to benefit other plant species due to pollinator spillover (Morandin and Kremen 2013; Blaauw and Isaacs 2014; Riedinger et al. 2015). However, plants flowering simultaneously may also compete for pollinators (Pauw 2013). Even within the day, temporal niche differentiation could reduce competition and may thus be a structuring force of plant–pollinator networks, while flexibility in the timing of plants and pollinators may lead to temporally variable network structure (Spiesman and Gratton 2016; CaraDonna and Waser 2020).

In this study, we performed a field experiment to test whether the diel dynamics of plant–pollinator interactions are driven by the early flower closure of Cichorieae (a plant tribe in the Asteraceae family) as has been hypothesized by Fründ et al. (2011). Cichorieae usually close their flowers by noon, but can delay flower closure until the evening in response to lack of pollination (Fründ et al. 2011). Early flower closure of Cichorieae was observed to cause pollinators that visit Cichorieae in the morning to switch to *Achillea millefolium* in the afternoon (Fründ et al. 2011), suggesting that changes in the availability of one plant type may be relevant for the flower visitation of other plant species. In addition, pollinators specialized on Cichorieae (e.g., *Lasioglossum villosulum* and *Panurgus calcaratus*, Westrich 1989) were never observed in the afternoon (Fründ et al. 2011). However, it is not clear whether the limited foraging time of these species was due to an intrinsic activity pattern or was simply driven by the abundance of their preferred resource. Using an experimental approach, which is still rare among network studies (Dormann et al. 2017), allowed us to better evaluate the mechanisms driving such diel dynamics of plant–pollinator interactions.

For our experiment we made use of the pollination-dependent plastic flower closure of two common species of Cichorieae (*Crepis capillaris* and *Leontodon hispidus*) and excluded pollinators in the morning to make their flowers available in the afternoon. Cichorieae are attractive for a wide range of pollinators and were observed to take a central role in plant–pollinator networks (Maia et al. 2019). The manipulation of the flower availability of Cichorieae is thus suitable to study the effect of prolonged resource availability at the network level. By comparing manipulated networks with Cichorieae available both in the morning and afternoon to control networks with Cichorieae available only in the morning, our experiment allowed us to understand the importance of early flower closure for network structure and dynamics and to identify the flexible response of networks to altered timing of flower availability.

Specifically, we addressed three questions: (1) are diel dynamics of plant–pollinator interactions driven by the time-restricted availability of Cichorieae, or by intrinsic activity rhythms of pollinators? Due to the prominent role of Cichorieae in the plant–pollinator network we expected that at least some generalist pollinators will make use of Cichorieae available in the afternoon, but that intrinsic rhythms of pollinators prevent the full usage of those additional resources in the afternoon. (2) Do diel dynamics in the interactions between Cichorieae and their pollinators influence temporal dynamics of interactions between other plants and their pollinators, potentially related to competition or facilitation? We expected that some pollinators that visit Cichorieae in the morning switch to other plants in the afternoon, and that delayed flower closure weakens such a pattern, reducing visitation to other plants in the afternoon. (3) How does the afternoon availability of Cichorieae flowers affect the structure of the full-day network? We expected that our treatment decreases network specialization as the extended flower availability may accumulate more (generalist) pollinators on Cichorieae and increase resource use overlap in the network.

## Methods

### Experimental design

The experiment was conducted at three sites (Freiburg, 48° 00′ 50.1″ N 7° 50′ 10.4″ E; Ebnet, 47° 58′ 59.9″ N 7° 55′ 21.9″ E; Kirchzarten, 47° 57′ 44.3″ N 7° 56′ 36.7″ E) close to the city of Freiburg, Germany, in July and August 2018. All sites had a high density of Cichorieae (either *Crepis capillaris* or *Leontodon hispidus*) in flower at this time. These herbs belong to the Asteraceae family and have yellow flowers arranged in composite flower heads (capitula) that were found to close in response to pollination but typically around noon (Fründ et al. 2011). Given their high abundance and their open flower morphology (short corollas, easily accessible pollen), Cichorieae are visited by a wide range of pollinator species (Fründ et al. 2011; Maia et al. 2019). The Freiburg site was a dry grassland on public green space not mown before July or August. This site was dominated by *Crepis capillaris* as our focal species*,* with *Thymus vulgaris*, *Daucus carota*, *Pimpinella saxifraga*, and *Achillea millefolium* as other abundant species in flower (see Supplementary Table 2 for full species list). The two other sites (Ebnet and Kirchzarten) were hay meadows mown once a year in June or July and afterwards dominated by *Leontodon hispidus* as our focal species. Other abundant plant species in flower at these sites were *Centaurea jacea*, *Knautia arvensis*, and *Leucanthemum vulgare.* At two sites, there were very low numbers of one or two other species of the Cichorieae (*Hypochoeris radicata* at Freiburg and Ebnet and *Leontodon autumnalis* at Freiburg, Supplementary Table 2) that showed similar flower closure and visitation patterns as the two focal species.

At each site we carried out two experimental runs, which consisted of a reference day without application of a treatment and a consecutive treatment day during which we manipulated afternoon availability of Cichorieae flowers by excluding pollinators in the morning (Supplementary Figs. 1, 2, 3). We established ten 2 m × 1 m plots at each site, and assured that each plot contained a similar and relatively high number of flower heads of the focal Cichorieae species. Sites were separated by a minimum distance of 3 km, while plots within each site were arranged as five pairs of a control and a treatment plot (1–5 m distance between paired plots and 5–30 m distance between pairs).

On reference days, pollinator visits to all plant species were recorded on all plots during four sampling rounds (two in the morning, two in the afternoon). After all sampling was finished for the reference day, half of the plots were covered by pollinator-exclusion cages constructed from insect protection nets (RANTAI Hobbynetze, TYP S48), which had a height of approximately 1 m (Supplementary Fig. 2). The pollinator-exclusion treatment was applied until noon of the subsequent day (treatment days). On this second day, the five control plots without pollinator-exclusion cages were again sampled two times in the morning and two times in the afternoon, while the treatment plots were sampled two times in the afternoon after the pollinator-exclusion cages had been removed. The exact time of cage removal was adjusted based on the time when most Cichorieae flower heads in the control plots had already closed and thus was accomplished between 11:00 and 12:30. This 2-day sampling procedure (a “run”) was carried out twice at each site. Experimental runs at the same site were at least 5 days apart and plots were assigned to a different treatment in both runs to avoid any influence of plot identity.

### Data collection

During each of the four sampling rounds, two observers recorded pollinator visits to flowers within plots, resulting in two separate data sets. The first observer recorded each pollinator visiting a flower for 6 min per plot. These 6 min were pure observation time excluding the time necessary to catch and label insects. Individuals were identified in the field, or caught with a sweep net, killed with ethyl acetate and stored in dry tubes for later identification. All captured insect specimens were identified to species or morphospecies level with the help of taxonomists (see Acknowledgements). This procedure revealed the identities of pollinator species visiting the different plant species, but may have underestimated visitation rates as the individuals caught were likely to have visited more than one flower per plot. Thus, during each sampling round each plot was additionally observed by a second observer for 5 min to assess the total number of flower visits, including multiple visits by the same pollinator individual (here pollinators were not caught and, in most cases, not identified to species-level). For individuals with a very high number of visits we estimated the number of visits in the field or assigned a plausible number in retrospect and, if possible, based on observations of the same species (Supplementary Table 1). Although only pollinators that touched the reproductive organs of flowers were recorded, we highlight that observed pollinator visits may not equally contribute to the reproduction of plants (King et al. 2013). During each sampling round we also counted the numbers of open Cichorieae flower heads in each plot to assess whether the treatment had been effective. Flower heads were considered to be open if their diameter reached at least 50% of the diameter of a fully opened flower head.

We tested the effects of treatment, time of day, and sampling day on the number of Cichorieae flower heads as well as on the number of pollinator visits (counted by the second observer) to both Cichorieae and other plants with three generalized linear mixed models. As we did not have morning data for treatment plots on treatment days, we used three different subsets of the full data set for these models: (i) to show the effect of our treatment, we used only afternoon data and tested the effects of treatment, day, and the interaction of both on flower head abundance and number of visits. (ii) To show the effect of time of day, we used only control data and tested the effects of time of the day, sampling day, and the interaction of both on the three response variables. (iii) To show that there was no systematic plot bias between treatment and control plots, we used only reference day data and tested the effects of time of the day, treatment, and the interaction of both on the three response variables. In all cases, data were modeled on a negative binomial distribution using the “nbinom2” family in the glmmTMB package ver. 1.0.2.1 (Brooks et al. 2017) in R version 4.0.2 (R Core Team 2020).

### Construction of full-day networks

For each sampling day (6 reference days and 6 treatment days), we constructed two full-day plant–pollinator interaction networks, one for the control and one for the treatment. For constructing these networks, we considered only interaction data for which the species (or morphospecies) was identified (= data sampled by the first observer). Each network was based on interaction data sampled during 120 min pure observation time (4 rounds × 5 plots × 6 min). Control networks were based on all interactions observed in the five control plots across the four sampling rounds per day. Treatment networks were based on the interactions observed in control plots during the morning (on treatment days treatment plots were covered by nets during the morning), combined with all interactions observed in treatment plots during the afternoon. For treatment days, treatment networks thus represent an artificial scenario where Cichorieae flowers are available throughout the day. The “treatment” networks for reference days were constructed in the same way as for treatment days (combining morning data from control plots and afternoon data from treatment plots) to allow comparisons.

### Timing flexibility of pollinators

To test whether flexibility in timing depends on the degree of specialization of pollinators, we classified pollinator species with > 5 observations as Cichorieae specialists if those species visited Cichorieae in > 90% of cases. We performed a paired t-test to test whether the contribution of these specialists to the observed visits on Cichorieae differs between morning and afternoon in treatment networks.

### Temporal network dynamics

To describe the diel dynamics of plant–pollinator interactions underlying the full-day networks, we constructed morning and afternoon sub-networks for each day and calculated four measures of dissimilarity between the two sub-networks: plant turnover, pollinator turnover, link turnover, and link rewiring. These four measures of temporal dynamics were calculated (using the bipartite package ver. 2.14 in R: Dormann et al. 2009; Fründ 2021) as quantitative Jaccard dissimilarities between sub-networks, which were standardized to proportions beforehand. Plant and pollinator turnover reflect temporal dissimilarities between communities derived from marginal totals. Link turnover (betaWN) is the total dissimilarity between the two sets of interactions. Link rewiring refers to switching of interaction partners over time among temporally co‐occurring species (Poisot et al. 2012) and thus only considers the dissimilarity of interactions among species shared between morning and afternoon sub-networks. We focused on differences between morning and afternoon as flower heads of Cichorieae usually close around noon.

### Pollinator sharing and switching

To explore the influence of our treatment on pollinator sharing and switching among Cichorieae and other plants, we assigned the visits observed in the afternoon to three categories: (a) pollinators that had visited Cichorieae in the morning; (b) pollinators that had visited other plants in the morning, and (c) pollinators that were not observed in the morning (due to pollinator timing or undersampling). For each pollinator species, we first assessed the relative frequency of Cichorieae and other plant species, respectively, among its morning visits to define the degree (proportion) to which it belonged to category (a) and (b), respectively. Second, we multiplied the afternoon visits for each pollinator species by the proportion it belonged to (a) and (b), respectively, to assign afternoon visits to these categories. If a pollinator species was only observed in the afternoon, we assigned all its visits to category (c), i.e., pollinators entering the network in the afternoon. Afternoon visits assigned to each of the three categories were then summed across pollinator species separately for Cichorieae and other plants.

### Network structure

To compare the structure of full-day networks between control and treatment, we calculated plant generality, pollinator generality (Bersier et al. 2002), modularity Q (Beckett 2016), and network specialization H_2_’ (Blüthgen et al. 2006). These network indices were used to understand how temporal availability of Cichorieae flowers affects different aspects of specialization in networks. Weighted quantitative generality can be calculated for trophic levels (plants and pollinators) and reflects the mean effective number of interaction partners of the species in the focal group weighted by their marginal totals (Bersier et al. 2002). To understand which part of the network drives the overall change, plant generality was also calculated separately for Cichorieae only (Cichorieae generality) and for all plants excluding Cichorieae (non-Cichorieae generality). Modularity Q describes the degree of compartmentalization in a network and ranges from 0 (the network does not have more links within modules than expected by chance) to a maximum value of 1 (all links are within modules) (Olesen et al. 2007; Beckett 2016). Network specialization H_2_’ describes the degree of specialization among plants and pollinators within the network (Blüthgen et al. 2006) and ranges between 0 (extreme generalization) and 1 (extreme specialization). We expected that extended flower availability of Cichorieae in our treatment will accumulate more pollinators on Cichorieae, thereby increasing plant generality, in particular Cichorieae generality. We also expected mostly generalist pollinators to respond to extended availability of Cichorieae, thereby increasing pollinator generality. In addition to this overall expectation of more generalized networks in the treatment, we expected that extended availability of Cichorieae will reduce temporal niche differentiation and increase connectivity between network compartments, and thus reduce modularity and H_2_’.

### Null model comparisons and permutation tests

To assess the significance (“non-randomness”) of the observed values of our response variables describing network dynamics and structure, we compared the mean of observed values across the six experimental runs against a corresponding null model. For each null model simulation, we constructed one random treatment and one random control network for each of the six runs, calculated the respective response variable and averaged over these six values to generate one null model mean. We repeated this procedure 1000 times to provide 95% confidence intervals for the mean of the null model values. This procedure was the same for all null model comparisons although the null models differed slightly between response variables.

The null model for the measures of temporal dynamics (“timing null model”) randomly shuffled interactions (visits) among morning and afternoon sub-networks but kept constant the frequency of interactions per sub-network as well as the frequency of each unique link per day. It thus tested whether the observed values were simply due to abundance differences between morning and afternoon or due to real differences in the timing of interactions.

To test for the significance of network structure and elucidate the influence of species timing on network structure, we compared the observed network indices against two null models. These null models randomized interactions within networks but accounted for (i) the abundance per plant and pollinator species (“structure null model”) and (ii) the abundance per plant and pollinator species per time of the day (“structure-per-time null model”). The structure null model was realized by applying the Patefield algorithm (function r2table called by function nullmodel in bipartite) to the full-day networks. The structure-per-time null model applied the Patefield algorithm separately to morning and afternoon sub-networks and afterwards summed both sub-networks to generate a randomized full-day network. Using the Patefield algorithm, which keeps row and column sums constant, allowed us to account for species abundances. The only difference between these two null models is whether or not network structure is constrained by species timing.

To test whether response variables differed between treatment and control networks, we performed permutation tests that compared the observed difference between treatment and control against the difference between two sets of random combinations of study plots irrespective of their true treatment. We first generated all 252 possible combinations of five plots from our ten study plots, and subsequently drew one of these 252 plot combinations for each sampling day. All plots within a drawn combination were used to construct randomized treatment networks, while the remaining plots were used to construct randomized control networks. Note that we again used the morning data of the five true control plots for both randomized treatment and randomized control networks. We finally calculated the difference between these randomized control and treatment networks for each sampling day and, separately for reference and treatment days, took the mean across all six runs. We repeated this procedure 5000 times and calculated the 95% confidence interval for the mean difference between networks of randomized plot combinations. An observed mean difference outside the confidence interval indicates a significant effect of the treatment.

## Results

### Flower abundance and flower visitation

**Fig. 1:**
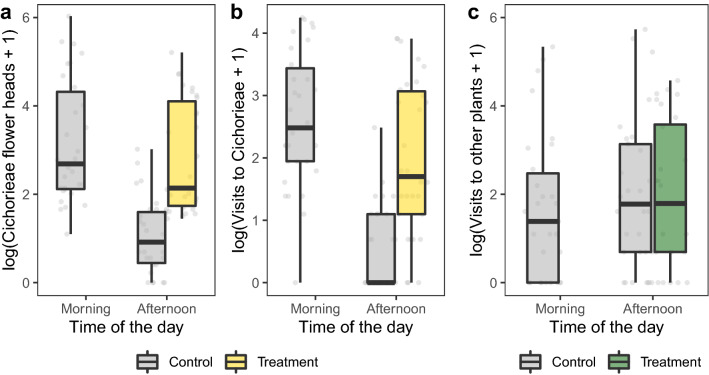
Effects of time of the day and treatment on **a** flower head abundance of Cichorieae; **b** pollinator visits to Cichorieae, and **c** pollinator visits to other plants on treatment days. Data were log-transformed. There are no data of treatment plots in the morning as plots were covered by pollinator-exclusion cages

During all six experimental runs, pollinator exclusion on treatment plots in the morning was effective in maintaining high abundance of open Cichorieae flower heads in the afternoon (Fig. 1a, Table 1). As a result, afternoon abundance of Cichorieae flower heads in treatment plots was only slightly lower than the flower head abundance observed during the morning in control plots (Fig. 1a). In contrast, for control plots that were accessible for pollinators in the morning, we found sharp declines in Cichorieae flower head abundance from the morning to the afternoon (Fig. 1a, Supplementary Table 3a). Depending on the sampling day, our treatment appeared to be fully effective only until 2–3 pm so that many flower heads in treated plots were already closed in the fourth sampling round.

**Table 1:** Effects of treatment (pollinator exclusion in the morning), sampling day (reference day vs. treatment day), and interaction of both on Cichorieae flower head abundance, the number of pollinator visits to Cichorieae, and the number of pollinator visits to other plants

Model	Df	*X*^2^	Pr(> *X*^2^)
Cichorieae flower abundance ~			
Treatment	1	38.68	** < 0.001**
Day	1	52.68	** < 0.001**
Treatment × day	1	35.29	** < 0.001**
Visits to Cichorieae ~			
Treatment	1	33.85	** < 0.001**
Day	1	64.18	** < 0.001**
Treatment × day	1	6.11	**0.013**
Visits to other plants ~			
Treatment	1	0.19	0.661
Day	1	1.09	0.297
Treatment × day	1	0.14	0.705

Here we used only afternoon data as our experiment did not allow for sampling treatment plots in the morning of treatment days. Significant effects are reported in bold

Similarly, we found that our treatment caused unusually high numbers of pollinator visits to Cichorieae flowers in the afternoon (Fig. 1b, Table 1). In control plots, the number of pollinator visits to Cichorieae was high in the morning but decreased to almost zero in the afternoon, when most flower heads had closed. This decrease in pollinator activity was slightly less pronounced on treatment days (Supplementary Table 3a). Flowers of other plant species received a similar number of visits in the afternoon in both control and treatment plots as in the morning in control plots (Fig. 1c, Table 1, Supplementary Table 3a). During reference days, treatment and control plots were indistinguishable in terms of flower head abundance of Cichorieae, pollinator visits on Cichorieae, and pollinator visits on other plants, indicating no systematic plot bias (Supplementary Fig. 4, Supplementary Table 3b). For the number of pollinator individuals on Cichorieae and other plants (data collected by the first observer and used for network analyses), we found exactly the same pattern (Supplementary Fig. 5).

### Timing flexibility of pollinators

**Fig. 2:**
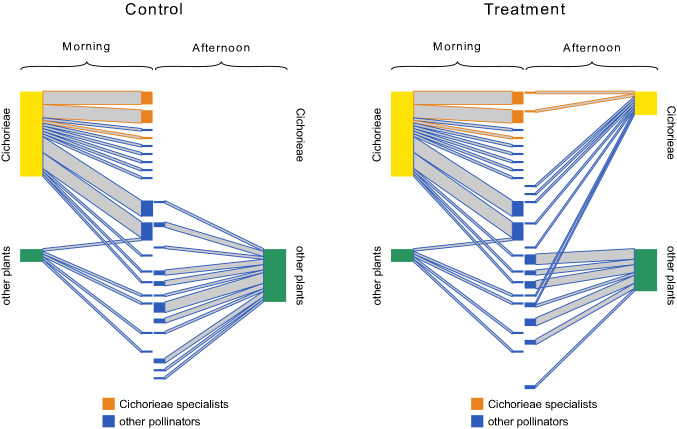
Paired morning and afternoon sub-networks that were constructed for control and treatment on treatment days. As an example for the whole experiment, this shows the first experimental run at the site “Kirchzarten” (see Supplementary Fig. 8–10 for all runs). For graphical reasons plants were pooled into two groups (Cichorieae and other plant species) and pollinator species were sorted according to their specialization on Cichorieae and their abundance. Pollinator species that were identified as Cichorieae specialists (> 90% of visits to Cichorieae and > 5 observed visits) are represented by orange boxes. The orientation is plant–pollinator for morning sub-networks and pollinator–plant for afternoon sub-networks. Pollinator boxes of sub-networks are aligned based on species identity to visualize species turnover and abundance differences. Box widths scale with the number of visits per plant group or pollinator species

At the study level we could identify six pollinator species specialized on Cichorieae: *Panurgus calcaratus*, *Eupeodes corollae*, *Andrena flavipes*, *Lasioglossum malachurum*, *L. villosulum*, and *L. leucozonium*. Two of these species are specialists of Cichorieae according to the literature (*P. calcaratus* is oligolectic, *L. villosulum* has a strong preference, Westrich 1989), for two others (*L. malachurum* and *L. leucozonium*) a strong preference has recently been detected (Wood et al. 2016), and the other two species are generalists that showed specialization in this study. In treatment plots (but not in controls), we observed some of these specialists also in the afternoon, when their preferred resource normally is not available (Fig. 2, Supplementary Figs. 8, 9, 10). The relative contribution of these specialists to the observed visits on Cichorieae did not differ between morning and afternoon in treatment networks (15.7% vs. 20.2%; *t* = 0.430, df = 5, *p* value = 0.69).

### Temporal network dynamics

The effects of our treatment also became visible at the network level: while control networks contained no afternoon interactions with Cichorieae in five of six cases, all treatment networks contained multiple afternoon interactions between Cichorieae and different pollinators (Fig. 2, Supplementary Figs. 8, 9, 10).

**Fig. 3:**
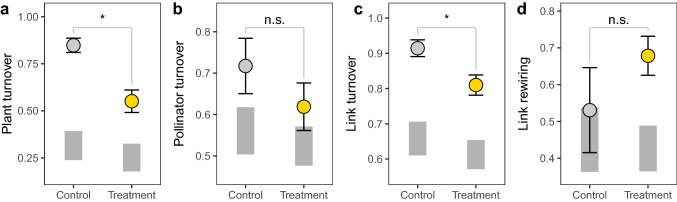
Treatment (extended Cichorieae flower availability) effects on diel turnover of **a** plant species, **b** pollinator species, **c** links, and **d** shared links (rewiring). Turnover was assessed as quantitative Jaccard dissimilarity between morning and afternoon sub-networks that were standardized to proportions. Means and standard errors are based on six experimental runs. Significant differences between observed means (treatment vs. control) are indicated by asterisks (*) and were inferred from permutation tests. Grey boxes represent 95% confidence intervals of the timing null model that randomly shuffles interactions among morning and afternoon sub-networks and keeps the frequency of interactions constant within sub-networks

Permutation tests revealed that our treatment significantly affected diel network dynamics (Fig. 3, see Supplementary Fig. 6 for dynamics on reference days). Plant turnover and link turnover decreased significantly in the treatment, but there were no effects on pollinator turnover and link rewiring (Fig. 3, Supplementary Table 4). Comparisons against the timing null model revealed for all days and treatments that turnover of plant and pollinator species as well as of their links was significantly greater than expected by chance (Fig. 3a–c, Supplementary Table 5). Thus, diel dynamics of the plant–pollinator networks at our study sites were not the result of undersampling. However, for control networks, link rewiring was within the range suggested by the timing null model and only for treatment networks there was significant rewiring (Fig. 3d, Supplementary Table 5).

When using only data from sampling round 3 for the afternoon sub-network in the assessment of diel turnover, we found exactly the same results, indicating that diel turnover was not caused by the imperfect treatment in sampling round 4 (Supplementary Fig. 7). Although treatment networks represent a combination of morning control and afternoon treatment plots, we did not find any significant treatment effect on diel turnover on reference days (Supplementary Fig. 6), suggesting that our results were not driven by differences in species composition between control and treatment plots.

### Pollinator sharing and switching

**Fig. 4:**
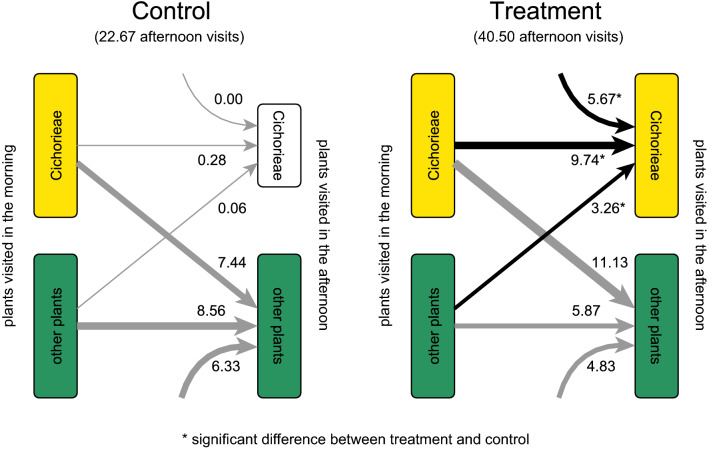
Origin of pollinators visiting Cichorieae and other plants in the afternoon of treatment days: did they visit Cichorieae or other plants, or were they not observed in the morning? The total number of afternoon visits to Cichorieae or other plant species was assigned to pollinators visiting either Cichorieae, other plant species, or no plants in the morning based on the relative frequency of Cichorieae and other plant species in the morning visits of individual pollinator species. The white Cichorieae box (control) indicates absence or low number of Cichorieae flowers in the afternoon. Numbers indicate and arrow widths scale with the mean number of visits across the six experimental runs. Curved arrows represent visits of pollinators that were not observed in the morning. Note that we observed 1.8 times as many afternoon visits in the treatment than in the control. Asterisks (*) and arrows colored in black indicate a significant difference between treatment and control based on permutation tests

Based on permutation tests, the treatment had positive effects on afternoon visits to Cichorieae by pollinators that were not observed in the morning, pollinators that had visited Cichorieae in the morning, and pollinators that had visited other plants in the morning (Fig. 4, Supplementary Tables 4, 6). In contrast, the number of afternoon visits to other plants was not significantly affected by the treatment, irrespective of whether pollinators were not observed, visited other plants, or visited Cichorieae before (Fig. 4, Supplementary Tables 4, 6).

### Network structure

**Fig. 5:**
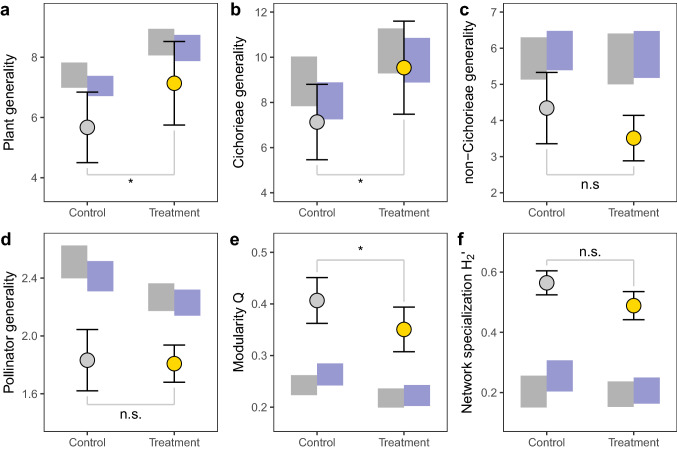
Treatment (extended Cichorieae flower availability) effects on network indices: **a** plant generality, **b** Cichorieae generality, **c** non-Cichorieae generality, **d** pollinator generality, **e** modularity Q, and **f** network specialization H_2_’. Only results for treatment days are presented. Means and standard errors are based on six experimental runs. Significant differences between means are indicated by asterisks (*) and were inferred from permutation tests. Grey boxes represent 95% confidence intervals of the structure null model that randomizes networks while keeping the frequency of interactions per plant and pollinator species constant. Blue boxes represent 95% confidence intervals of the structure-per-time null model that only randomizes interactions within the morning and afternoon sub-networks while keeping the frequency of interactions per plant and pollinator species and per time of the day constant

Our treatment had only limited influence on the structure of full-day networks (Fig. 5, Supplementary Fig. 11, Supplementary Tables 4, 7). The treatment increased plant generality through its positive effect on the generality of Cichorieae, while generality of other plants and pollinator generality were not significantly affected by the treatment. The treatment decreased modularity Q, but had no effect on network specialization H_2_’. Overall, network indices of both control and treatment networks were well in the range typically observed for full-day networks (Schwarz et al. 2020). In both control and treatment networks, plant generality (including generality of Cichorieae and other plants) and pollinator generality were significantly lower than predicted by both the structure and the structure-per-time null models, while modularity and network specialization were significantly higher than predicted by the two null models (Fig. 5, Supplementary Table 7). This shows that networks were significantly specialized independent of the treatment. For pollinator generality, both null models predicted a reduction with the treatment, but such a change was not found for the observed values (Fig. 5d). The structure-per-time null model was always closer to the observed values than the structure null model that did not account for time of the day. However, overall both null models yielded quite similar ranges and the null model considering time of the day was still far from the observed values (Fig. 5).

## Discussion

Using a novel experimental approach, we show that the early flower closure of Cichorieae around noon is indeed an important driver of diel network dynamics. When Cichorieae flower heads are experimentally kept open in the afternoon, they are visited by a wide range of pollinators, indicating that the pollinator community as a whole is flexible enough to make use of these additional resources. Thus, both plants and pollinators can adjust their timing in response to the other trophic level: plants shorten their flowering time when there are enough pollinators, while pollinators extend their activity time when given a reason to do so. Remarkably, we could not find evidence for increased competition for pollinators between Cichorieae and other species in response to delayed flower closure of Cichorieae. The effect of our treatment on network structure and dynamics was mainly the result of increased availability of Cichorieae and less due to distinct temporal niches of pollinators.

### Drivers of temporal network dynamics (question 1)

The results of our experiment complement and confirm the findings of Fründ et al. (2011), who hypothesized that early flower closure of Cichorieae is a significant driver of diel dynamics of plant–pollinator networks. We found that under natural conditions the drastic reduction of Cichorieae flower availability in the afternoon leads to an almost complete loss of interactions with Cichorieae, which explains high link turnover (= interaction dissimilarity) between morning and afternoon. In contrast, if we prevent early flower closure, Cichorieae flowers available in the afternoon are visited in similar numbers as those in the morning. Thus, in our treatment many Cichorieae interactions persisted in the afternoon, thereby reducing link turnover. However, as link turnover was significant even in the treatment, there appear to be temporal dynamics independent of resource availability, which could be explained by species-specific activity patterns of pollinators (Herrera 1990; Wiggam and Ferguson 2005; Knop et al. 2018). In fact, we found significant diel pollinator turnover in both treatment and control. However, the treatment did not significantly alter pollinator turnover, which suggests that increased turnover due to new species in the afternoon and reduced turnover due to re-sampling more old species may have resulted in no net effect on pollinator turnover. Given the network-level approach of our study, we are unable to identify the species-specific patterns and drivers of pollinator timing, which overall appeared less important for network dynamics than the temporal change in flower availability. Our treatment also did not affect link rewiring, i.e., a change in which pollinators visit which flowers. Nevertheless, significant link rewiring at the whole-network level (only found for treatment networks) indicates some flexibility in partner choice among plants and pollinators.

Among the pollinators found on Cichorieae in the afternoon were also species that appeared to be specialized on Cichorieae in our study. Thus, the normally early end of the foraging activity of these specialist pollinators (Fründ et al. 2011) may be rather determined by scarcity of resources than by an internal clock or external drivers of pollinator activity, including temperature (McCall and Primack 1992; Rader et al. 2013; Knop et al. 2018) or enemies (Lienhard et al. 2010). Considering that weather can be quite variable in Central Europe even within a day, pollinator species should show some degree of flexibility allowing them to forage at different times during the day. In contrast, a fixed daily activity rhythm is more likely to evolve in more predictable and extreme environments, e.g., in deserts, where foraging might be restricted to the morning and the evening to avoid overheating and desiccation (Willmer and Stone 1997; Gottlieb et al. 2005). Likewise, very cold temperatures limiting bee activity (Stone 1994) were also not important in our networks (studied in summer). Still, pollinator turnover was significantly different from our null model in both treatment and control, suggesting that distinct activity times of pollinators, potentially linked to variation in thermal niches (Kühsel and Blüthgen 2015) or internal clocks (Bloch et al. 2017), contribute to diel dynamics of plant–pollinator networks.

Overall, our study highlights that timing of flower resource availability is a key driver of diel dynamics of plant–pollinator interactions (see also Stone et al. 1999; Carvalho et al. 2013). Although interactions newly formed in the afternoon, due to either rewiring or pollinator turnover, may add to diel dynamics, interactions lost in the afternoon due to the early flower closure of Cichorieae appear to be more important in driving diel dynamics in networks where this group of plants is dominant. Importantly, our results also show that temporal network dynamics do not result from independent timing of plants and pollinators (Herrera 1990), but are predominantly driven by responses of plants and pollinators to the respective other trophic level (Stone et al. 1999). Cichorieae respond to lack of pollination by delaying their flower closure, and pollinators respond readily to an increased afternoon availability of Cichorieae flowers by visiting those flowers. Such adaptability in timing implies that temporal network dynamics themselves can be subject to change. Thus, while the strong effect of Cichorieae flower closure confirms that network dynamics can be predicted by knowledge of diel or seasonal phenology, fixed species timing assumptions will misrepresent the expected dynamics. Taken together, our results highlight that temporal dynamics of plant–pollinator interactions can be observed within a few hours and thus should be considered in the sampling and analysis of plant–pollinator networks even at short temporal scales.

### Pollinator sharing and switching (question 2)

Our analyses suggest that there is only limited influence of the early flower closure of Cichorieae on potential indirect effects between Cichorieae and other plants. We did not find evidence for increased competition between Cichorieae and other plants as increased afternoon availability of Cichorieae flowers in our treatment had no effect on the total number of visits to other plants. Our findings rather indicate the potential for facilitative effects of Cichorieae when available in the afternoon. Although our treatment increased switching of pollinators from other plants in the morning to Cichorieae in the afternoon, it tended to increase switching from Cichorieae in the morning to other plants in the afternoon to an even larger degree (although the latter trend was not significant). Thus, Cichorieae available in the afternoon may attract visitors to other plants rather than luring them away, indicating some potential for synchronous facilitation (Ghazoul 2006; Lázaro et al. 2009, 2014). Although we cannot rule out the possibility that our treatment (pollinator exclusion in the morning) increased nectar and pollen availability also of other plants, such a direct treatment effect on other plants likely played no major role as afternoon visitation by the typical visitors of these other plants did not increase. We evaluated only visitation patterns here, thus leaving the possibility that our treatment increased competition through heterospecific pollen deposition (Morales and Traveset 2008). Due to the large flight ranges of pollinators, performing replicated experimental manipulations on the optimal scale is challenging. In our study, plots were much smaller than pollinator foraging ranges. Future experiments on larger spatial scales would be useful for robust conclusions about pollinator-mediated indirect effects among plants (cf. Riedinger et al. 2015). Overall, our results caution against simple conclusions about competition derived from aggregate network patterns: metrics based on resource overlap and interaction frequency only indicate a “potential for indirect effects” (Carvalheiro et al. 2014). In contrast, the explicit consideration of the temporal dimension of plant–pollinator networks may allow for a better understanding of indirect effects in networks (Baldock et al. 2011).

### Network structure (question 3)

The effect of experimentally delayed flower closure on network structure was mainly driven by the increased availability of Cichorieae in the network and only weakly affected by the timing of interactions. Extending Cichorieae flower availability to the afternoon led to increased plant generality, which reflects an increase in the number of interaction partners of Cichorieae but not of other plants. As both null models (“structure” and “structure-per-time”) yielded such effects, the increase in plant generality appeared to be mostly due to the increased number of visits to Cichorieae in our treatment. Cichorieae generality was relatively high compared to non-Cichorieae generality and values for plant generality reported in other studies (Schwarz et al. 2020), which highlights the importance of Cichorieae as food resource of many pollinator species and may explain their central role in plant–pollinator networks (Maia et al. 2019). Both null models predicted a decrease in pollinator generality in the treatment, likely reflecting that fewer plant species may be used per pollinator if the dominance of one plant species is increased (Bersier et al. 2002). However, we did not find such decrease in pollinator generality, likely because an increased attraction of generalist pollinators or increasingly generalized behavior (mixing between Cichorieae and other plants in the afternoon) counterbalanced the predicted negative effect on pollinator generality. Overall, this confirms that Cichorieae are generalists in the pollination network and could interact with even more different pollinator species if they keep their flowers open in the afternoon.

Modularity decreased in response to delayed flower closure in our treatment although the associated increase in network size typically has the opposite effect (Olesen et al. 2007). This decrease in modularity could be explained by a combination of lost temporal structure and increased Cichorieae dominance, both of which may increase the overlap in pollinator species between Cichorieae and other plants and thus lead to more connected network compartments. Although the increase in plant generality and the decrease in modularity indicate that networks became less specialized in the treatment, we found no effect of our treatment on network specialization H_2_’. As H_2_’ is standardized between minimum and maximum values possible for given marginal totals, it controls for the effect of increasing Cichorieae dominance. The effect of lost temporal structure alone may not have been strong enough for finding a significant effect on H_2_’. Nevertheless, all indices (except Cichorieae generality) were significantly different from our null models (assuming no specialization of species), which indicates a significant degree of specialization in networks with and without Cichorieae available in the afternoon. Our null model comparisons also revealed that diel timing of plant–pollinator interactions contributed only slightly to the observed structure of plant–pollinator networks. Similarly, for a Brazilian bee–flower network it was found that bees separate their niches more strongly in resource use than in diel foraging time (Santos et al. 2013). Thus, the effects of diel timing on network structure seem to be mostly driven by temporal availability of abundant species determining overall interaction frequencies and only little by temporal matching of plant and pollinator species.

## Conclusions

Our experiment confirms the idea that temporally restricted availability of floral resources is a key driver of diel dynamics of plant–pollinator interactions. We show that the early flower closure of Cichorieae leads to the almost complete loss of interactions between Cichorieae and their pollinators in the afternoon, explaining high diel link turnover. These temporal dynamics occur within a few hours, which underlines the importance of considering time in sampling and analysis of networks even at short temporal scales. Our results also suggest effects of temporal flower availability on pollinator activity need to be considered for indirect interactions between plants, given that we did not find evidence for increased competition despite increased synchrony. Importantly, we find that diel temporal dynamics are not a fixed network characteristic but are shaped by flexibility in both timing and partner choice of the species forming the network. This flexibility may allow plant and pollinator species to fulfill their functional roles within a network despite environmental variability at short temporal scales and may thus facilitate long-term network stability.

## Supplementary Information

Below is the link to the electronic supplementary material.

Supplementary file1 (PDF 4249 kb)

Supplementary file2 (PDF 82 kb)

Supplementary file3 (CSV 117 kb)

Supplementary file4 (CSV 380 kb)

Supplementary file5 (R 64 kb)

Supplementary file6 (R 380 kb)

## Data Availability

All data generated or analyzed during this study are included in this published article [and its supplementary information files].

## Code availability

The code used to generate the results of this study is included in this published article [and its supplementary information files].
