# Supplementary material for: Within-day dynamics of plant–pollinator networks are dominated by early flower closure: an experimental test of network plasticity
Source: Oecologia. 2021 Jun 3;196(3):781–94. doi: 10.1007/s00442-021-04952-5 (PMC8292255; doi:10.1007/s00442-021-04952-5)
Supplement: Supplementary file 1 — Supplementary file1 (PDF 4249 kb) [file 442_2021_4952_MOESM1_ESM.pdf]

Supplementary material

Within-day dynamics of plant-pollinator networks are dominated by early flower closure: an experimental test of network plasticity

Benjamin Schwarz[1], Carsten F. Dormann[1], Diego P. Vázquez[2,3], Jochen Fründ[1]

[1] Biometry and Environmental System Analysis, University of Freiburg, Tennenbacher Str. 4, 79106 Freiburg, Germany

[2] Argentine Institute for Dryland Research, CONICET, Av. Ruiz Leal s/n, 5500 Mendoza, Argentina

[3] Faculty of Exact and Natural Sciences, National University of Cuyo, Padre Jorge Contreras 1300, M5502JMA Mendoza, Argentina

Supplementary Figures

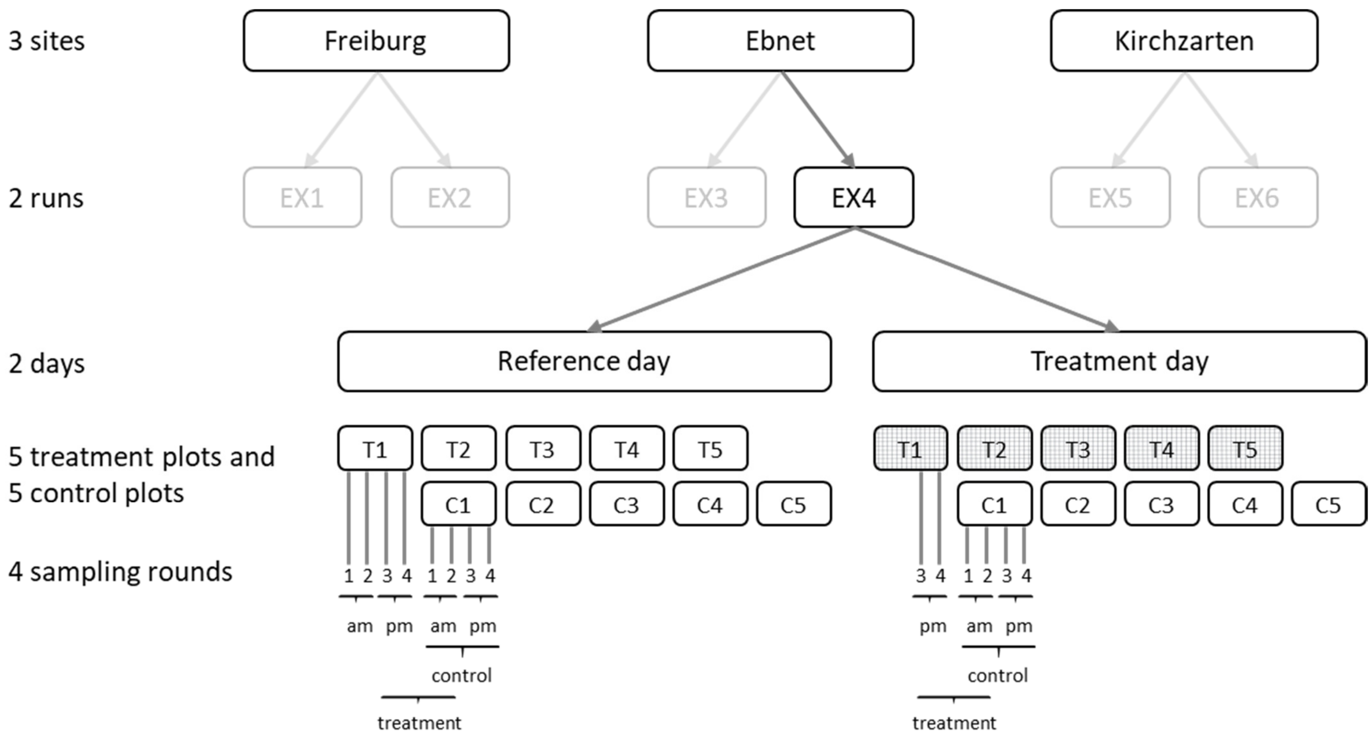

Supplementary Figure 1. Experimental design. For analyses at the network level all observations were pooled by treatment and day but across plots. As on treatment days treatment plots were not sampled in the morning (am), full-day treatment networks were constructed by pooling interactions observed in the morning in control plots and interactions observed in the afternoon in treatment plots.

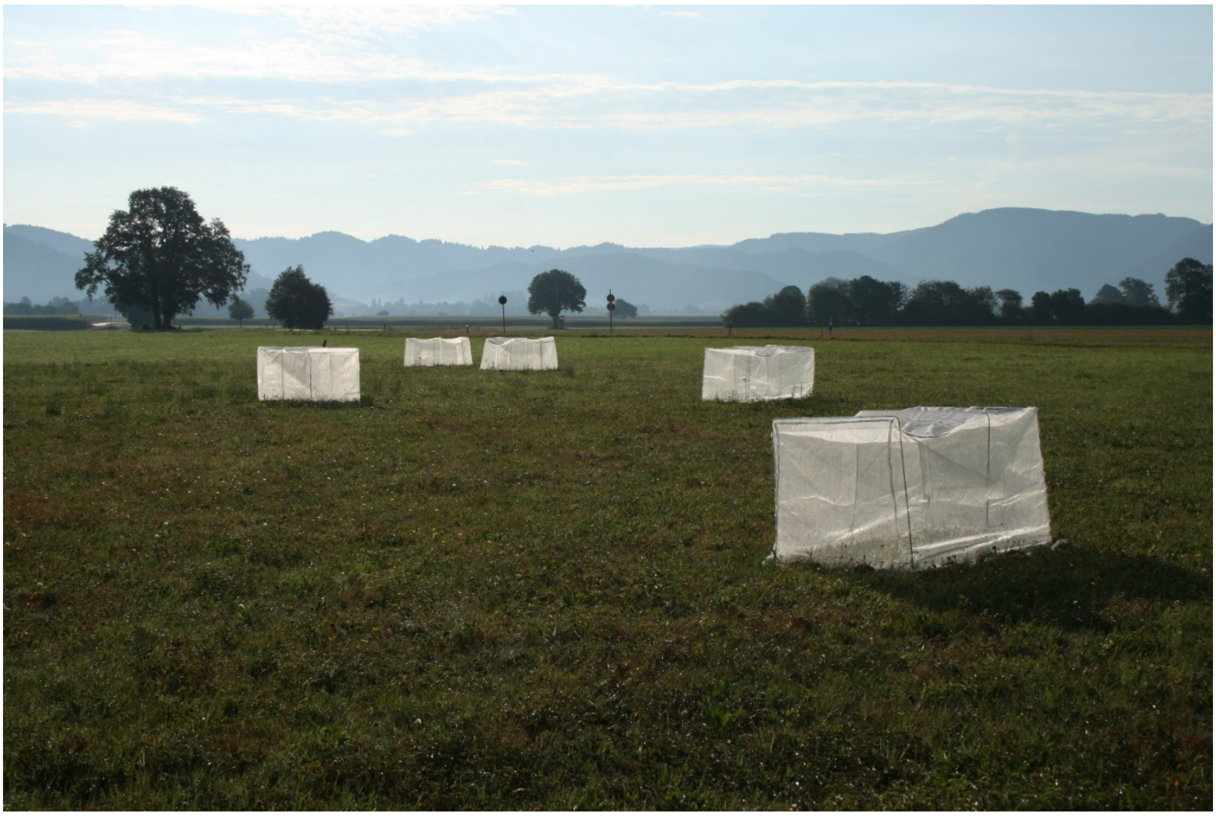

Supplementary Figure 2. Five pollinator exclusion cages at the site “Ebnet” (run 2 at 02-Aug- 2018, 08:11). Photo: Benjamin Schwarz.

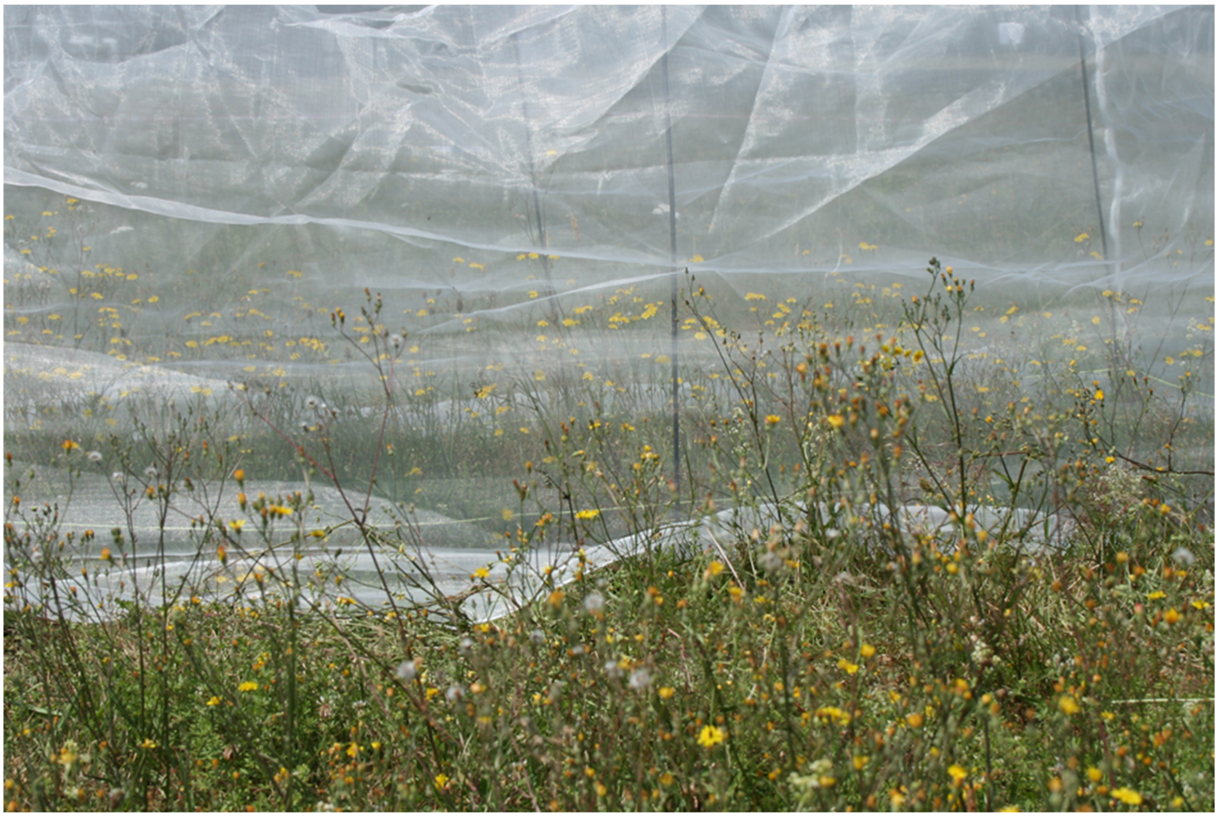

Supplementary Figure 3. Flowers of *Crepis capillaris* open to pollinators (foreground) and covered by a pollinator exclusion cage (background) at the site “Freiburg” (run 1 at 02-Jul-2018, 12:45). The photo was taken shortly before the cage was removed. At this time most flower heads outside the cage had already been closed, while they were still fully open inside the cage. Photo: Benjamin Schwarz.

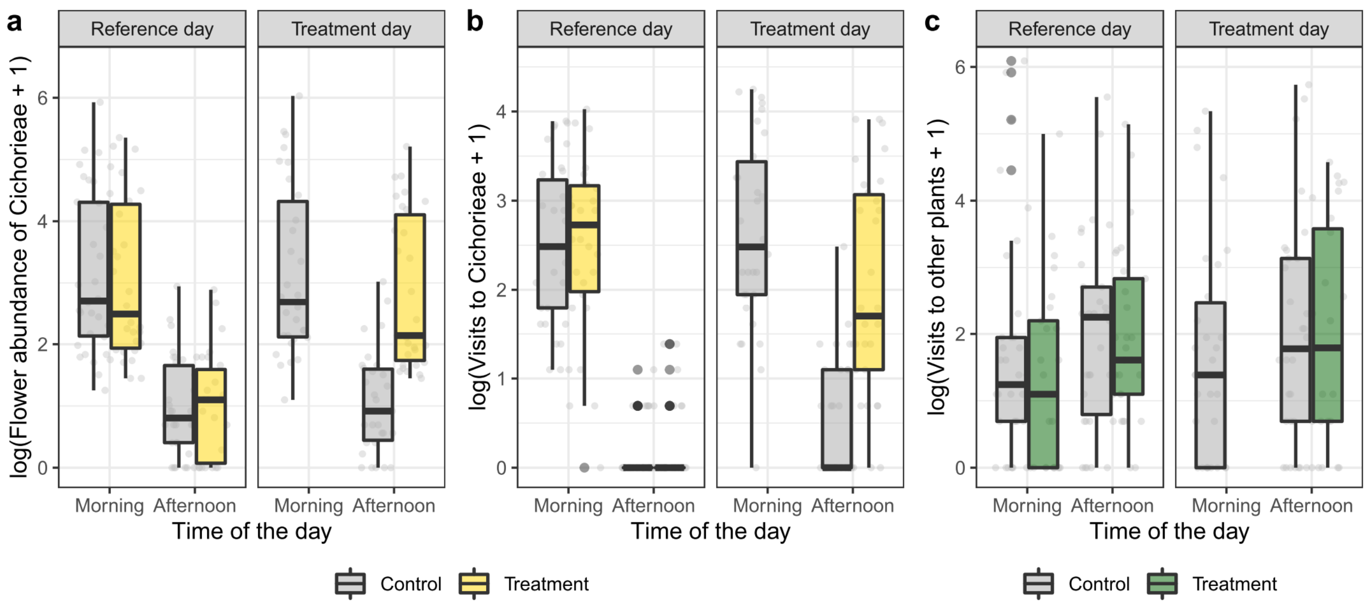

Supplementary Figure 4. Effects of day, time of the day, and treatment on (a) flower abundance of Cichorieae, (b) pollinator visits to Cichorieae, and (c) pollinator visits to other plants. Data were log-transformed. There are no data of treatment plots in the morning of treatment days as plots were covered by pollinator exclusion cages. This figure is equivalent to Fig. 1 in the main text but complemented by reference days.

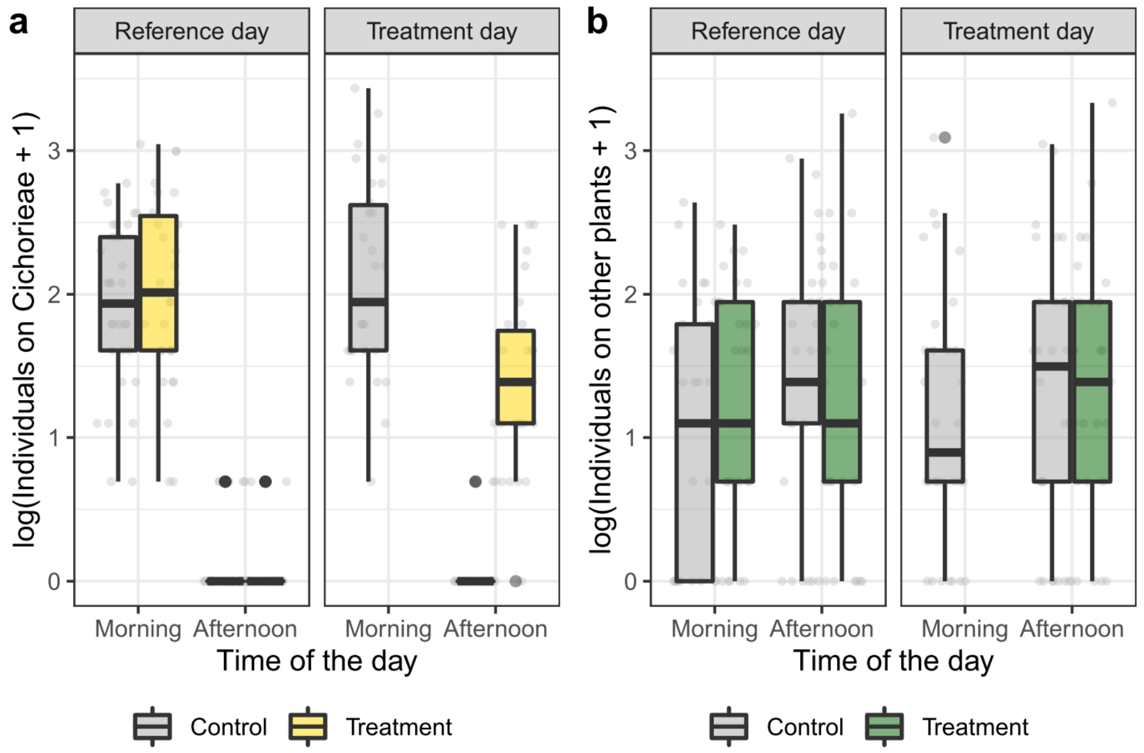

Supplementary Figure 5. Effects of day, time of the day, and treatment on (a) observed pollinator individuals on Cichorieae, and (b) observed pollinator individuals on other plants. Data were log-transformed. There are no data of treatment plots in the morning of treatment days as plots were covered by pollinator exclusion cages. In contrast to Fig. 1 b-c in the main text and Supplementary Figure 3 b-c, here the number of pollinator individuals and not the total number of flower visits is presented while the pattern is the same in both cases.

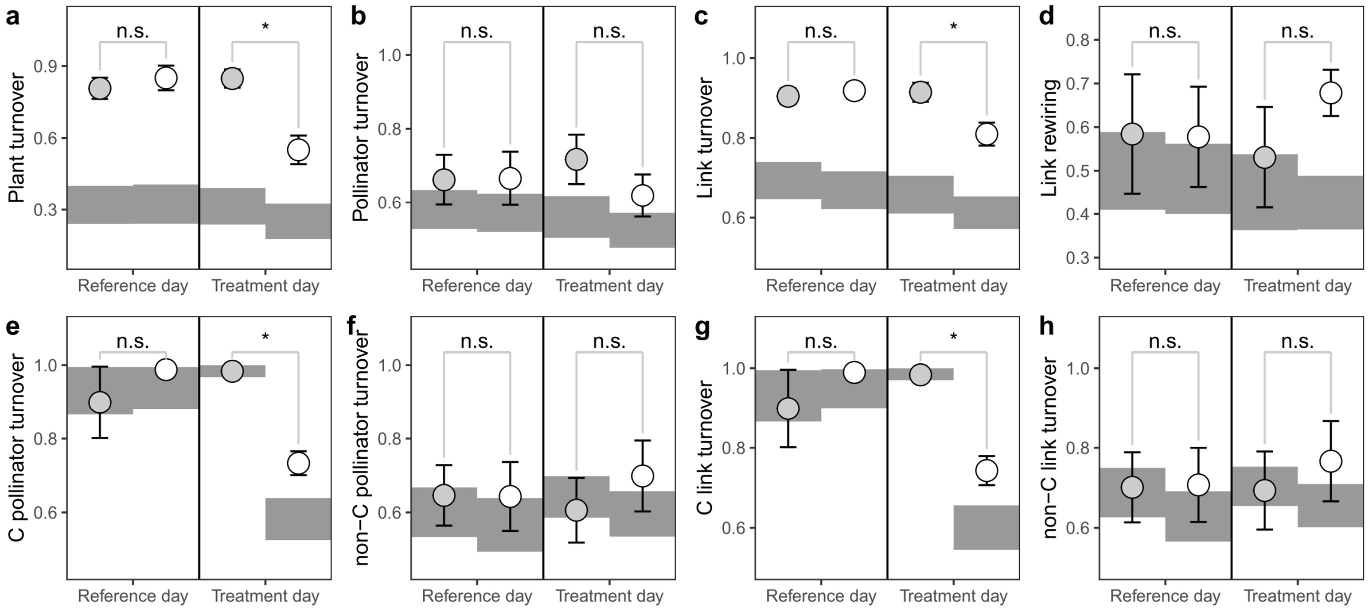

Supplementary Figure 6. Effects of day and treatment (control: grey circles; treatment: white circles) on diel turnover of plant species, pollinator species, links, and shared links (rewiring) assessed for the complete network (a-d) as well as on pollinator and link turnover assessed for the Cichorieae (e, g) and non-Cichorieae sub-networks (f, h). Turnover was assessed as quantitative Jaccard dissimilarity between morning and afternoon sub-networks that were standardized to proportions. Means and standard errors are based on six experimental runs. Significant differences between means are indicated by asterisks (*) and were inferred from permutation tests. Grey boxes represent 95% confidence intervals of the timing null model that randomly shuffles interactions among morning and afternoon sub-networks and keeps the frequency of interactions constant within sub-networks. Panels a-d are equivalent to Fig. 3 in the main text but complemented by reference days.

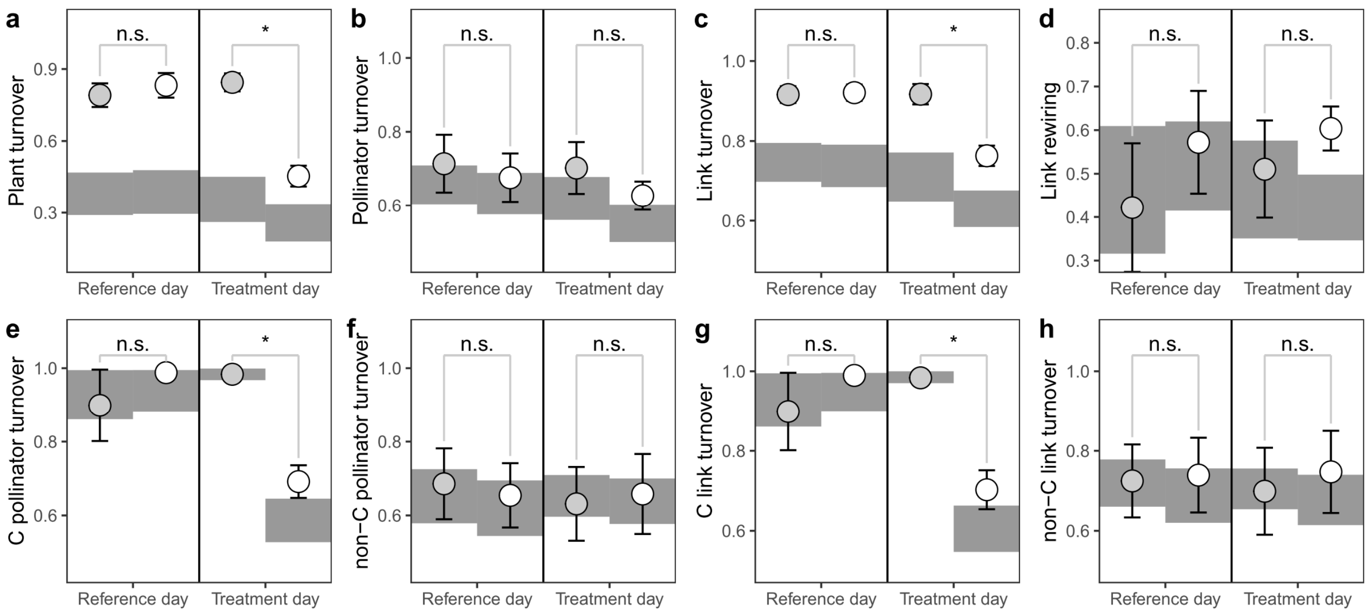

Supplementary Figure 7. Effects of day and treatment (control: grey circles; treatment: white circles) on diel turnover of plant species, pollinator species, links, and shared links (rewiring) assessed for the complete network (a-d) as well as on pollinator and link turnover assessed for the Cichorieae (e, g) and non-Cichorieae sub-networks (f, h). Turnover was assessed as quantitative Jaccard dissimilarity between morning and afternoon sub-networks that were standardized to proportions. In contrast to Fig. 3 in the main text and Supplementary Fig. 6, here we used only data from sampling round 3 for the afternoon sub-network to test whether turnover was caused by the imperfect treatment in sampling round 4 (some Cichorieae flowers already were closed in this round). Means and standard errors are based on six experimental runs. Significant differences between means are indicated by asterisks (*) and were inferred from permutation tests. Grey boxes represent 95% confidence intervals of the timing null model that randomly shuffles interactions among morning and afternoon sub-networks and keeps the frequency of interactions constant within sub-networks.

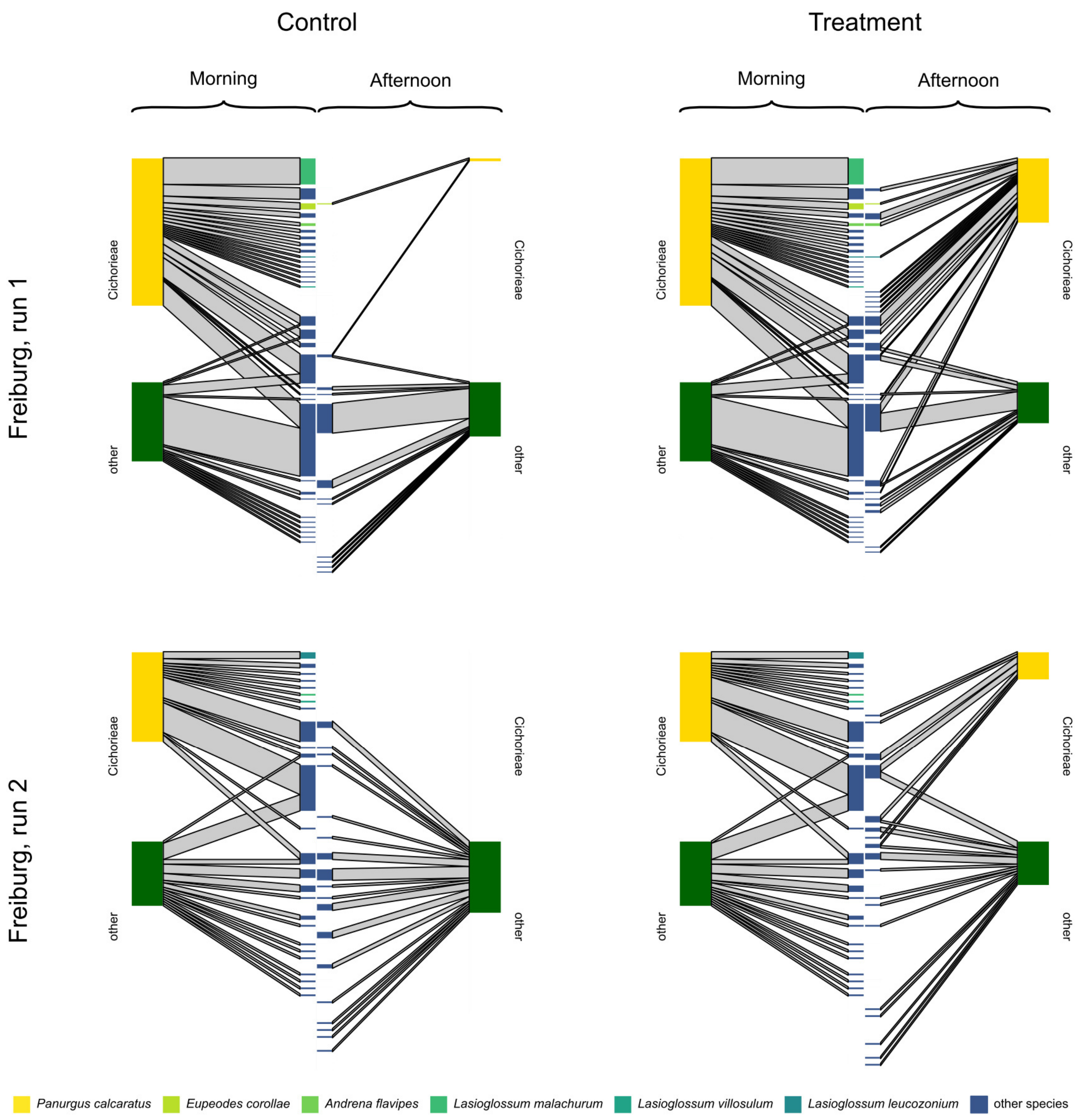

Supplementary Figure 8. Paired morning and afternoon sub-networks that were constructed for control and treatment on treatment days of the two experimental runs at the site "Freiburg". For graphical reasons plants were pooled into two groups (Cichorieae and other plant species) and pollinator species were sorted according to their specialization on Cichorieae and their abundance. Six pollinator species were identified as Cichorieae specialists (>90% of visits to Cichorieae and >5 observed visits in total), which can be identified by the color of boxes. The orientation is plant-pollinator for morning sub-networks and pollinator-plant for afternoon sub-networks and pollinator boxes of sub-networks were aligned based on species identity to visualize species turnover and abundance differences. Box widths scale with the number of visits per plant group or pollinator species.

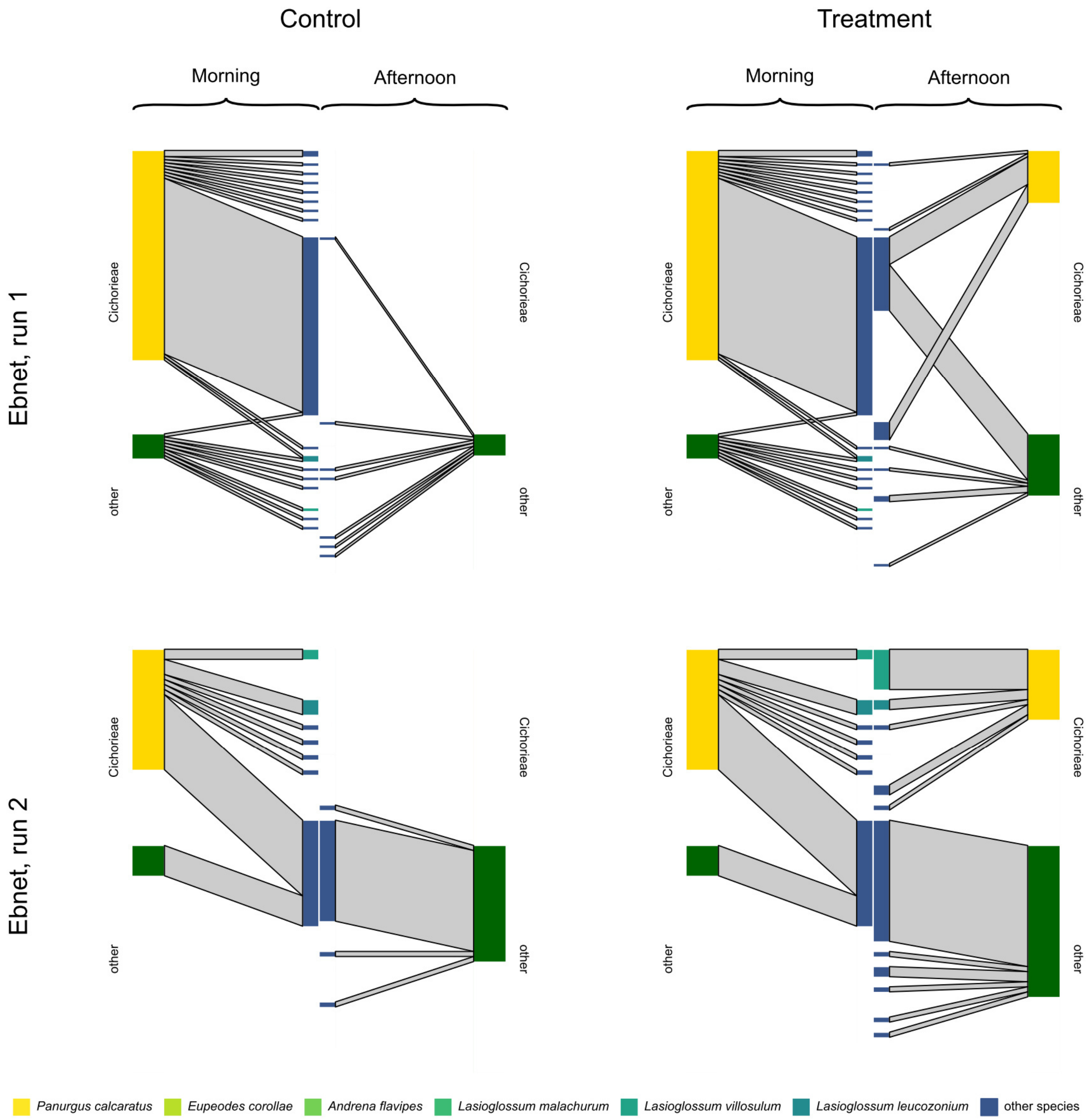

Supplementary Figure 9. Paired morning and afternoon sub-networks that were constructed for control and treatment on treatment days of the two experimental runs at the site "Ebnet". For graphical reasons plants were pooled into two groups (Cichorieae and other plant species) and pollinator species were sorted according to their specialization on Cichorieae and their abundance. Six pollinator species were identified as Cichorieae specialists (>90% of visits to Cichorieae and >5 observed visits in total), which can be identified by the color of boxes. The orientation is plant-pollinator for morning sub-networks and pollinator-plant for afternoon sub-networks and pollinator boxes of sub-networks were aligned based on species identity to visualize species turnover and abundance differences. Box widths scale with the number of visits per plant group or pollinator species.

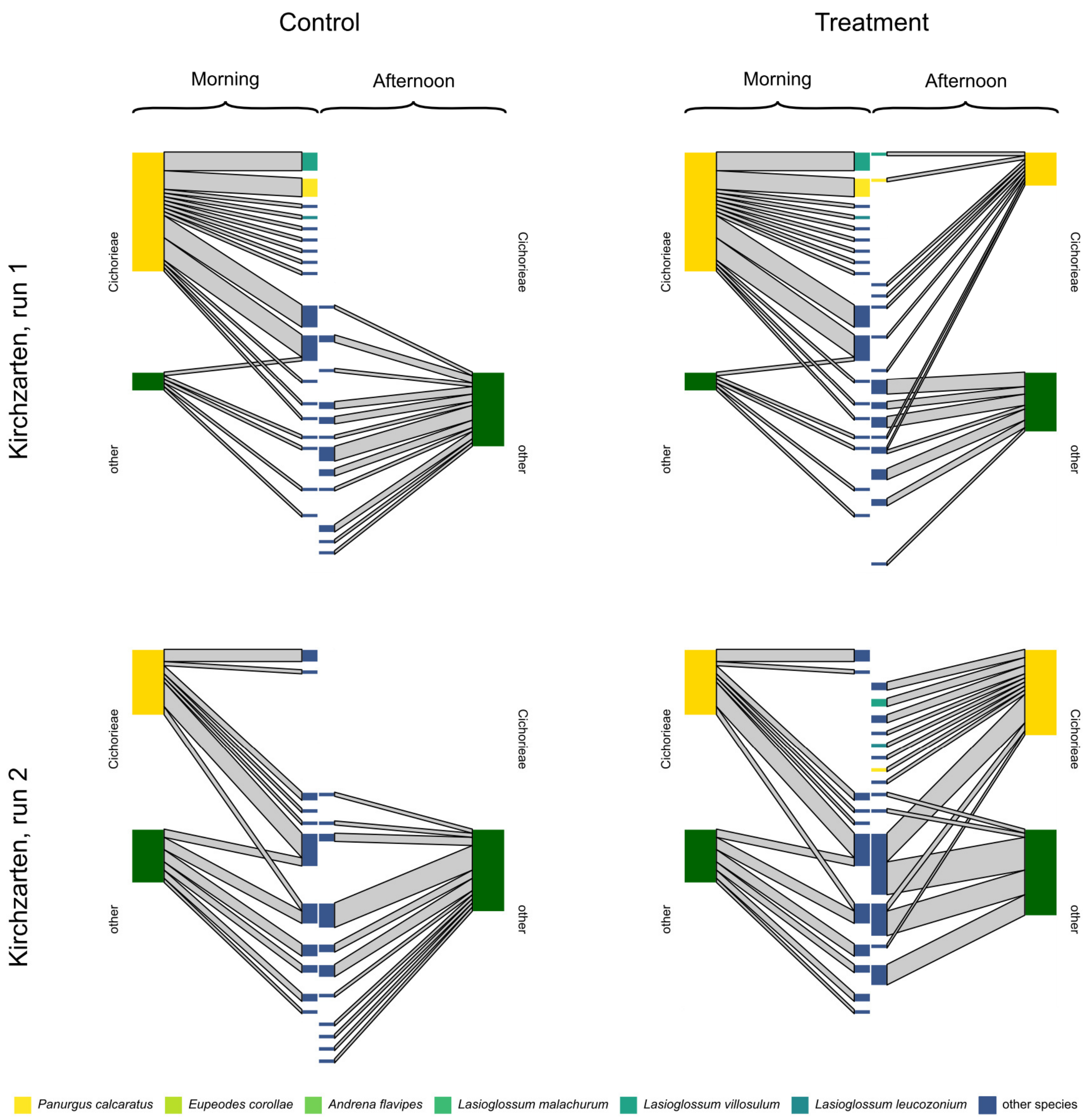

Supplementary Figure 10. Paired morning and afternoon sub-networks that were constructed for control and treatment on treatment days of the two experimental runs at the site "Kirchzarten". For graphical reasons plants were pooled into two groups (Cichorieae and other plant species) and pollinator species were sorted according to their specialization on Cichorieae and their abundance. Six pollinator species were identified as Cichorieae specialists (>90% of visits to Cichorieae and >5 observed visits in total), which can be identified by the color of boxes. The orientation is plant-pollinator for morning sub-networks and pollinator-plant for afternoon sub-networks and pollinator boxes of sub-networks were aligned based on species identity to visualize species turnover and abundance differences. Box widths scale with the number of visits per plant group or pollinator species.

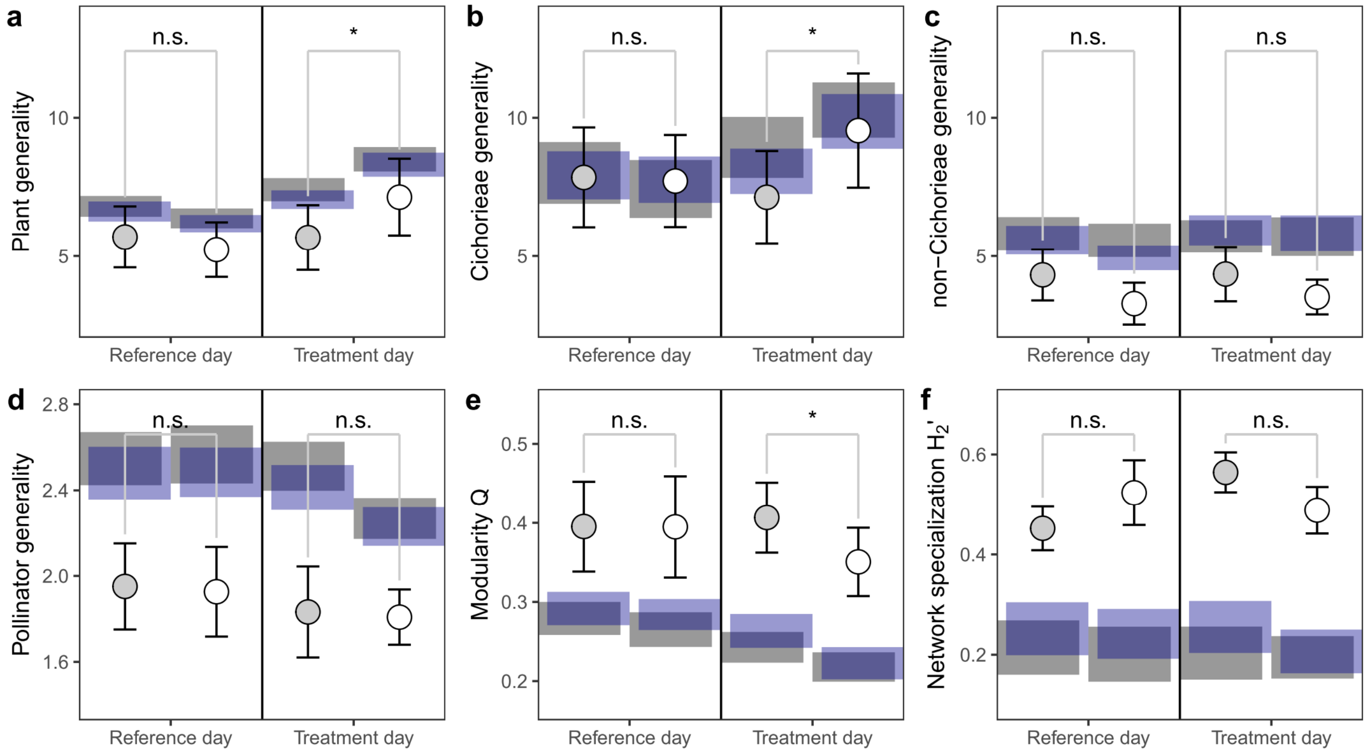

Supplementary Figure 11. Effects of day and treatment (control: grey circles; treatment: white circles) on (a) plant generality, (b) Cichorieae generality, (c) non-Cichorieae generality, (d) pollinator generality, (e) modularity Q, and (f) network specialization H_2'. Means and standard errors are based on six experimental runs. Significant differences between means are indicated by asterisks (*) and were inferred from permutation tests. Grey boxes represent 95% confidence intervals of null models that randomize networks while keeping the frequency of interactions per plant and pollinator species constant (r2d algorithm). Blue boxes represent 95% confidence intervals of null models that only randomize interactions within the morning and afternoon sub-networks while keeping the frequency of interactions per plant and pollinator species and per time of the day constant. This figure is equivalent to Fig. 5 in the main text but complemented by reference days.

Supplementary Tables

Supplementary Table 1. Number of visits that was assigned in retrospect if the exact number could not be observed in the field.

**Plant**	**Animal**	**Assigned number of visits**	**Condition**
Thymus vulgaris	*Apis mellifera*	35	insect was foraging for the whole observation period
Daucus carota	*Bombus terrestris*	25 per flower head	in the field only the number of flower heads visited was noted
Daucus carota	ants and bugs	5 per flower head	in the field only the number of flower heads visited was noted
Daucus carota	other insects	10 per flower head	in the field only the number of flower heads visited was noted
Pimpinella saxifraga	all insects	5 per flower head	in the field only the number of flower heads visited was noted

Supplementary Table 2. List of plant species present at the three experimental sites. Number of visits (assessed by the second observer) and number of network interactions (= number of pollinator individuals recorded by the first observer) are given as sums across all experimental runs, sampling days, and treatments per site and plant species. Plant species of the Cichorieae are marked in bold.

Site	Plant species	# Visits	# Network interactions
Freiburg	*Thymus vulgaris*	1882	147
Freiburg	*Daucus carota*	1428	55
Freiburg	***Crepis capillaris***	933	266
Freiburg	*Pimpinella saxifraga*	491	52
Freiburg	*Achillea millefolium*	100	22
Freiburg	*Plantago lanceolata*	92	25
Freiburg	*Trifolium repens*	64	18
Freiburg	*Galium mollugo*	45	18
Freiburg	*Senecio vulgaris*	37	9
Freiburg	*Trifolium pratense*	24	10
Freiburg	***Hypochoeris radicata***	17	11
Freiburg	*Trifolium arvense*	15	3
Freiburg	*Lotus corniculatus*	14	5
Freiburg	***Leontodon autumnalis***	3	1
Freiburg	*Stellaria sp.*	2	0
Freiburg	*Trifolium campestre*	0	0
Ebnet	***Leontodon hispidus***	915	198
Ebnet	*Centaurea jacea*	328	147
Ebnet	*Pimpinella saxifraga*	93	23
Ebnet	*Trifolium pratense*	21	9
Ebnet	*Galium mollugo*	7	0
Ebnet	***Hypochoeris radicata***	6	12
Ebnet	*Leucanthemum vulgare*	3	2
Ebnet	*Trifolium repens*	1	2
Ebnet	*Plantago lanceolata*	1	0
Ebnet	*Campanula sp.*	0	0
Ebnet	*Lotus corniculatus*	0	0
Ebnet	*Polygala vulgaris*	0	0
Ebnet	*Prunella vulgaris*	0	0
Kirchzarten	*Centaurea jacea*	243	115
Kirchzarten	***Leontodon hispidus***	230	117
Kirchzarten	*Knautia arvensis*	51	29
Kirchzarten	*Leucanthemum vulgare*	25	33
Kirchzarten	*Galium mollugo*	24	3
Kirchzarten	*Trifolium pratense*	1	2
Kirchzarten	*Trifolium repens*	0	1
Kirchzarten	*Lotus corniculatus*	0	0
Kirchzarten	*Plantago lanceolata*	0	0

Supplementary Table 3. Effects of a) time of the day, sampling day, and their interaction, and b) time of the day, treatment, and their interaction on Cichorieae flower abundance, the number of pollinator visits to Cichorieae, and the number of pollinator visits to other plants. Note that for a) and b) we used different subsets of the full data set, respectively, as our experiment did not allow to sample treatment plots in the morning of treatment days and thus to test interactions on the full data set.

Model	Chisq	Df	Pr(>Chisq)
**a) using only control plot data**			
Cichorieae flower abundance ~			
Time of the day	216.35	1	**<0.001**
Day	0.23	1	0.63
Time of the day × Day	0.02	1	0.899
Visits to Cichorieae ~			
Time of the day	212.43	1	**<0.001**
Day	5.87	1	**0.015**
Time of the day × Day	8.11	1	**0.004**
Visits to other plants ~			
Time of the day	0.05	1	0.825
Day	0.49	1	0.485
Time of the day × Day	0.1	1	0.752
**b) using only reference day data**			
Cichorieae flower abundance ~			
Time of the day	284.06	1	**<0.001**
Treatment	0.41	1	0.521
Time of the day × Treatment	0.02	1	0.893
Visits to Cichorieae ~			
Time of the day	212.43	1	**<0.001**
Treatment	5.87	1	0.735
Time of the day × Treatment	8.11	1	0.287
Visits to other plants ~			
Time of the day	2.19	1	0.139
Treatment	0.75	1	0.387
Time of the day × Treatment	2	1	0.158

Supplementary Table 4. Permutation test results indicating the significance of treatment effects on response variables describing temporal dynamics, pollinator sharing and switching, and network structure. If the observed difference between control and treatment was within the 95% confidence interval (CI), it was not significant (n.s.). If the difference was lower or greater than the 95% CI, the treatment had a significantly positive (↑) or negative (↓) effect, respectively.

**Response**	**Reference day**				**Treatment day**			
	**Observed difference [control - treatment]**	**Lower bound 95% CI**	**Upper bound 95% CI**	**Treatment effect**	**Observed difference [control - treatment]**	**Lower bound 95% CI**	**Upper bound 95% CI**	**Treatment effect**
**Temporal dynamics**								
Plant turnover	-0.043	-0.081	0.087	n.s.	0.297	-0.195	0.190	↓
Pollinator turnover	-0.004	-0.132	0.133	n.s.	0.098	-0.155	0.154	n.s.
Link turnover	-0.014	-0.035	0.035	n.s.	0.105	-0.067	0.066	↓
Link rewiring	0.006	-0.343	0.336	n.s.	-0.148	-0.153	0.156	n.s.
C link turnover	-0.091	-0.112	0.112	n.s.	0.241	-0.142	0.140	↓
C pollinator turnover	-0.089	-0.113	0.113	n.s.	0.250	-0.145	0.147	↓
nonC link turnover	-0.006	-0.144	0.145	n.s.	-0.073	-0.154	0.159	n.s.
nonC pollinator turnover	0.003	-0.151	0.152	n.s.	-0.093	-0.181	0.179	n.s.
**Pollinator sharing and switching**								
Cichorieae → Cichorieae	0.079	-0.579	0.579	n.s.	-9.458	-3.586	3.587	↑
other plants → Cichorieae	-0.246	-0.254	0.254	n.s.	-3.209	-1.725	1.679	↑
not observed → Cichorieae	0.167	-0.500	0.500	n.s.	-5.667	-2.667	2.667	↑
Cichorieae → other plants	-1.340	-7.868	7.736	n.s.	-3.691	-8.248	8.300	n.s.
other plants → other plants	1.007	-4.303	4.227	n.s.	2.691	-5.370	5.437	n.s.
not observed → other plants	1.667	-3.333	3.667	n.s.	1.500	-3.833	3.833	n.s.
**Network structure**								
Plant generality	0.461	-0.835	0.840	n.s.	-1.463	-0.837	0.854	↑
Cichorieae generality	0.131	-0.593	0.570	n.s.	-2.407	-1.019	0.995	↑
non-Cichorieae generality	1.042	-1.193	1.195	n.s.	0.832	-0.905	0.903	n.s.
Pollinator generality	0.025	-0.162	0.165	n.s.	0.024	-0.182	0.186	n.s.
Modularity Q	0.000	-0.045	0.044	n.s.	0.056	-0.053	0.053	↓
Network specialization H_2'	-0.072	-0.110	0.111	n.s.	0.076	-0.096	0.098	n.s.

Supplementary Table 5. Observed means and standard errors of temporal dynamics in comparison to the 95% confidence interval (CI) predicted by the timing null model. Means and standard errors are based on six experimental runs.

**Response**	**Day**	**Treatment**	**Mean**	**Standard error**	**Lower bound 95% CI**	**Upper bound 95% CI**	**Mean vs. CI**
Plant turnover	Reference day	Control	0.807	0.044	0.240	0.401	>
		Treatment	0.850	0.051	0.241	0.406	>
	Treatment day	Control	0.848	0.038	0.238	0.392	>
		Treatment	0.551	0.060	0.177	0.325	>
Pollinator turnover	Reference day	Control	0.662	0.068	0.527	0.634	>
		Treatment	0.666	0.072	0.519	0.624	>
	Treatment day	Control	0.717	0.067	0.504	0.618	>
		Treatment	0.619	0.058	0.477	0.571	>
Link turnover	Reference day	Control	0.904	0.020	0.647	0.740	>
		Treatment	0.918	0.020	0.621	0.717	>
	Treatment day	Control	0.915	0.024	0.610	0.706	>
		Treatment	0.810	0.029	0.571	0.654	>
Link rewiring	Reference day	Control	0.584	0.137	0.410	0.589	=
		Treatment	0.578	0.115	0.400	0.562	>
	Treatment day	Control	0.531	0.116	0.363	0.538	=
		Treatment	0.678	0.053	0.364	0.489	>
Cichorieae pollinator turnover	Reference day	Control	0.899	0.097	0.866	0.995	=
		Treatment	0.988	0.010	0.880	0.995	=
	Treatment day	Control	0.984	0.016	0.968	1.000	=
		Treatment	0.733	0.032	0.525	0.639	>
non-Cichorieae pollinator turnover	Reference day	Control	0.646	0.082	0.533	0.668	=
		Treatment	0.643	0.093	0.494	0.639	>
	Treatment day	Control	0.606	0.088	0.586	0.698	=
		Treatment	0.699	0.096	0.535	0.657	>
Cichorieae link turnover	Reference day	Control	0.899	0.097	0.866	0.995	=
		Treatment	0.990	0.010	0.900	0.998	=
	Treatment day	Control	0.984	0.016	0.970	1.000	=
		Treatment	0.743	0.036	0.545	0.656	>
non-Cichorieae link turnover	Reference day	Control	0.701	0.088	0.626	0.750	=
		Treatment	0.707	0.093	0.566	0.691	>
	Treatment day	Control	0.693	0.098	0.655	0.753	=
		Treatment	0.766	0.100	0.601	0.709	>

Supplementary Table 6. Observed means and standard errors of measures describing pollinator sharing and switching. Values represent the mean number of afternoon visits to Cichorieae and other plants by pollinators that had visited Cichorieae in the morning (Cichorieae →), pollinators that had visited other plants in the morning (other plants →), or pollinators that were not observed in the morning (not observed →). Means and standard errors are based on six experimental runs.

**Response**	**Day**	**Treatment**	**Mean**	**Standard error**
Cichorieae → Cichorieae	Reference day	Control	0.329	0.208
		Treatment	0.250	0.171
	Treatment day	Control	0.278	0.278
		Treatment	9.736	2.940
other plants → Cichorieae	Reference day	Control	0.004	0.004
		Treatment	0.250	0.250
	Treatment day	Control	0.056	0.056
		Treatment	3.264	1.624
not observed → Cichorieae	Reference day	Control	0.333	0.211
		Treatment	0.167	0.167
	Treatment day	Control	0.000	0.000
		Treatment	5.667	1.229
Cichorieae → other plants	Reference day	Control	6.651	1.684
		Treatment	7.991	2.969
	Treatment day	Control	7.439	1.801
		Treatment	11.131	1.799
other plants → other plants	Reference day	Control	7.849	2.771
		Treatment	6.842	1.576
	Treatment day	Control	8.561	2.248
		Treatment	5.869	1.712
not observed → other plants	Reference day	Control	7.000	1.612
		Treatment	5.333	1.563
	Treatment day	Control	6.333	1.820
		Treatment	4.833	1.167

Supplementary Table 7. Observed means and standard errors of network indices in comparison to the 95% confidence intervals (CI) predicted by the structure null model and the structure-per-time null model. Means and standard errors are based on six experimental runs.

Response	Day	Treatment	Mean	Standard error	Structure null model			Structure-per-time null model		
					Lower bound 95% CI	Upper bound 95% CI	Mean vs. CI	Lower bound 95% CI	Upper bound 95% CI	Mean vs. CI
Plant generality	Reference day	Control	5.695	1.106	6.429	7.181	<	6.253	6.979	<
		Treatment	5.235	0.989	6.009	6.727	<	5.865	6.489	<
	Treatment day	Control	5.673	1.171	6.988	7.825	<	6.711	7.382	<
		Treatment	7.136	1.387	8.061	8.943	<	7.873	8.743	<
Cichorieae generality	Reference day	Control	7.849	1.803	6.896	9.125	=	7.055	8.791	=
		Treatment	7.717	1.663	6.384	8.475	=	6.930	8.604	=
	Treatment day	Control	7.132	1.670	7.833	10.032	<	7.250	8.888	<
		Treatment	9.539	2.060	9.282	11.277	=	8.882	10.857	=
non-Cichorieae generality	Reference day	Control	4.316	0.927	5.205	6.415	<	5.059	6.097	<
		Treatment	3.273	0.758	4.965	6.177	<	4.484	5.387	<
	Treatment day	Control	4.345	0.987	5.131	6.302	<	5.391	6.478	<
		Treatment	3.513	0.629	5.000	6.407	<	5.177	6.477	<
Pollinator generality	Reference day	Control	1.951	0.200	2.424	2.671	<	2.357	2.602	<
		Treatment	1.926	0.209	2.432	2.702	<	2.369	2.599	<
	Treatment day	Control	1.832	0.212	2.398	2.626	<	2.308	2.518	<
		Treatment	1.808	0.129	2.172	2.364	<	2.140	2.321	<
Modularity Q	Reference day	Control	0.395	0.057	0.258	0.300	>	0.271	0.313	>
		Treatment	0.395	0.064	0.243	0.287	>	0.264	0.304	>
	Treatment day	Control	0.407	0.044	0.223	0.262	>	0.242	0.285	>
		Treatment	0.351	0.043	0.199	0.236	>	0.202	0.243	>
Network specialization H_2'	Reference day	Control	0.452	0.044	0.160	0.268	>	0.199	0.305	>
		Treatment	0.524	0.065	0.146	0.256	>	0.192	0.291	>
	Treatment day	Control	0.564	0.040	0.150	0.256	>	0.204	0.307	>
		Treatment	0.488	0.047	0.152	0.237	>	0.163	0.250	>
