# Supplementary material for: Within-day dynamics of plant–pollinator networks are dominated by early flower closure: an experimental test of network plasticity
Source: Oecologia. 2021 Jun 3;196(3):781–94. doi: 10.1007/s00442-021-04952-5 (PMC8292255; doi:10.1007/s00442-021-04952-5)
Supplement: Supplementary file 2 — Supplementary file2 (PDF 82 kb) [file 442_2021_4952_MOESM2_ESM.pdf]

README for data and R code

File list

interaction_data.csv

plotlevel_data.csv

Schwarz_et_al_Functions.R

Schwarz_et_al_Analyses.R

File description

interaction_data.csv – data set containing plant-pollinator interaction data recorded by the first observer. Needs to be red in Schwarz_et_al_Analyses.R to reproduce results. Plant species are listed in column “lower” and pollinator species in column “higher”.

plotlevel_data.csv – data set containing flower abundance of Cichorieae (column “focal.abun”) and the number of pollinator visits (“visits”) per plot recorded by the second observer. Needs to be red in Schwarz_et_al_Analyses.R to reproduce results.

Schwarz_et_al_Functions.R – R script containing functions that allow the computation of network indices and dissimilarity measures as well as performing null model comparisons and permutation tests.

Schwarz_et_al_Analyses.R – R script to run plot- and network-level computations and analyses.
